# Development of Zinc-Containing Chitosan/Gelatin Coatings with Immunomodulatory Effect for Soft Tissue Sealing around Dental Implants

**DOI:** 10.1007/s13770-024-00680-y

**Published:** 2024-11-23

**Authors:** Jing Han, Jorine G. F. Sanders, Lea Andrée, Bart A. J. A. van Oirschot, Adelina S. Plachokova, Jeroen J. J. P. van den Beucken, Sander C. G. Leeuwenburgh, Fang Yang

**Affiliations:** Department of Dentistry–Regenerative Biomaterials, Research Institute for Medical Innovation, Radboudumc, Philips Van Leijdenlaan 25, 6525 Nijmegen, The Netherlands

**Keywords:** Macrophage, Zinc ion, Chitosan, Gelatin, Soft tissue integration

## Abstract

*****BACKGROUND***:**

Soft tissue integration (STI) around dental implant abutments is a prerequisite to prevent bacterial invasion and achieve successful dental implant rehabilitation. However, peri-implant STI is a major challenge after dental abutment placement due to alterations in the immune microenvironment upon surgical dental implant installation.

*****METHODS***:**

Based on known immunomodulatory effects of zinc, we herein deposited zinc/chitosan/gelatin (Zn/CS/Gel) coatings onto titanium substrates to study their effect on macrophages. First, we exposed macrophages to cell culture media containing different zinc ion (Zn^2+^) concentrations. Next, we explored the immunomodulatory effect of Zn/CS/Gel coatings prepared via facile electrophoretic deposition (EPD).

*****RESULTS***:**

We found that Zn^2+^ effectively altered the secretome by reducing the secretion of pro-inflammatory and enhancing pro-regenerative cytokine secretion, particularly at a Zn^2+^ supplementation of approximately 37.5 μM. Zn/CS/Gel coatings released Zn^2+^ in a concentration range which effectively stimulated pro-regenerative macrophage polarization as demonstrated by M2 macrophage polarization. Additionally, the impact of these Zn^2+^-exposed macrophages on gingival fibroblasts incubated in conditioned medium showed stimulated adhesion, proliferation, and collagen secretion.

*****CONCLUSION***:**

Our promising results suggest that controlled release of Zn^2+^ from Zn/CS/Gel coatings could be applied to immunomodulate peri-implant STI, and to enhance dental implant survival.

## Introduction

Metallic implants serve diverse medical purposes, encompassing fields such as orthopedics, dentistry, and cardiology. In the field of dentistry, the annual application of around 15 million dental implants to replace missing teeth signifies their extensive use, offering patients with both fixed and removable prosthetic solutions with a focus on masticatory function and esthetics [1]. Traditionally, the improvement of dental implant quality has revolved around osseointegration, focusing on the integration between the implant and the surrounding alveolar bone. However, investigations into factors leading to implant failure reveal that peri-implant mucositis, an initial inflammation of the mucous tissues surrounding dental implants, is another crucial factor. Untreated peri-implant mucositis may progress to peri-implantitis, resulting in excessive bone resorption and eventually implant loss [2]. Therefore, achieving proper peri-implant soft tissue sealing, via both epithelial and connective tissues, is imperative to prevent bacterial invasion, enabling prolonged dental implant survival [3].

After surgery for dental implant installation, the tissue damage activates a host immune response in the tissues surrounding the dental implant. This immune response induces a series of reactions in the surrounding tissue, sequentially recruiting various immune cells to participate in the immunological process crucial for pathogen defense and tissue regeneration in wound healing [3, 4]. Previous research has identified diverse cytokines secreted by immune cells, including transforming growth factors (TGF), epithelial growth factor (EGF), and fibroblast growth factors (FGF), that support cell proliferation. Moreover, certain immune cells including neutrophils, macrophages, and dendritic cells can remove debris and secrete enzymes such as proteinase and collagenase, contributing to the reorganization of peri-implant tissue. However, a prolonged or uncontrolled immune response may lead to chronic inflammation, affecting the tissue repair, and eventually leading to impaired tissue integration [4, 5]. Therefore, it is of vital importance to create a pro-regenerative immune microenvironment to enhance tissue repair around dental implants.

A particularly promising approach to modulating the immune microenvironment involves bestowing implants with immunomodulatory properties, regulating immune cells to secrete pro-regenerative cytokines. Within the context of immunomodulation, macrophages are regarded as the most pivotal immune cells actively positioned at the interface between implant and tissue [6]. Specifically, macrophages exhibit notable plasticity, and upon activation, can polarize into either a pro-inflammatory phenotype (M1) or a pro-regenerative phenotype (M2) [7]. M1 macrophages typically secrete pro-inflammatory cytokines such as tumor necrosis factor (TNF)-α, interleukin (IL)-1β, and IL-6, exerting inhibitory effects on cell proliferation and inducing apoptosis. In contrast, M2 macrophages contribute to wound healing by producing cytokines and chemokines like TGF-β, IL-4, and IL-10, thereby promoting cell migration, proliferation, and extracellular matrix (ECM) synthesis [8]. Therefore, in the context of peri-implant STI, M2 macrophages exhibit a more favorable phenotype. The significant plasticity exhibited by macrophages renders them an ideal target for immunomodulatory modulation. Moreover, due to the long-lived property, macrophages participate in the process of tissue regeneration for prolonged time [9].

Surface engineering of implants to enhance tissue regeneration through immunomodulation has emerged as a compelling and alternative strategy [3, 4, 10]. Various studies confirm the feasibility of modulating the immune microenvironment by altering implant surfaces, a process achieved through modifications such as changes in surface topography or the application of coatings incorporating therapeutic agents [11]. These immunomodulatory approaches provide the possibility to influencing macrophage polarization, fostering an anti-inflammatory and pro-healing milieu conducive to tissue regeneration. Most of the previous immunomodulatory strategies have concentrated primarily on osteoimmunology, corresponding to the former predominant dental focus on osseointegration, the peri-implant bone integration [12]. However, STI is increasingly considered crucial to ensure long-term implant survival [13]. Consequently, in the context of STI, an urgent need exists to develop a platform capable of effectively attenuating initial acute inflammation following dental implant installation, promoting soft tissue regeneration, and concurrently preventing bacterial infection.

To module the distinctive immune microenvironment of peri-implant soft tissue, we propose a chitosan/gelatin-based coating incorporating zinc ions (Zn^2+^). Zinc plays a vital role in various key enzymes and transporters, contributing significantly to the immune system and wound healing [14, 15]. Controlled release of Zn^2+^ has been demonstrated to enhance the secretion of pro-regenerative cytokines by recruiting more M2 macrophages [16]. Notably, reported work exploring the immunomodulatory properties of zinc primarily focused on osteo-immunomodulation or skin healing, but studies on the effect of Zn^2+^ on peri-implant soft tissue are scarce [10, 16–18]. Moreover, it is not yet clear at which concentration range Zn^2+^ exerts therapeutic effects. To address this knowledge gap, we first cultured macrophages in Zn^2+^-containing media to identify the effective therapeutic concentrations of Zn^2+^ required to induce macrophage polarization into M2 macrophages. Subsequently, we prepared a zinc/chitosan/gelatin (Zn/CS/Gel) coating which can release Zn^2+^ in a sustainable and pH-responsive manner by means of electrophoretic deposition (EPD) to investigate the immunomodulatory potential of Zn^2+^ on macrophages by assessing their polarization. To investigate the immunomodulatory effect of Zn^2+^ on soft tissue, we investigated the effects of Zn^2+^-exposed macrophages on the behavior of gingival fibroblasts in terms of adhesion, proliferation, and collagen secretion. Our findings confirm the promising role of Zn^2+^ in immunomodulating peri-implant STI via a facile surface modification of dental implant abutments.

## Materials and methods

### Preparation of Zn^2+^-containing cell culture media

Zinc chloride (ZnCl_2_·6H_2_O, Sigma–Aldrich) was selected as the zinc source for this study. To prepare Zn^2+^-containing media, ZnCl_2_·6H_2_O was dissolved in RPMI 1640 medium (Gibco) to achieve a Zn^2+^ concentration of 6 mM. Subsequently, the Zn^2+^-containing medium was filtered using a 0.22 μm filter and stored at 4 °C as stock medium. For cell treatment, the stock medium was diluted to produce a series of concentrations using cell culture medium, resulting in final concentrations between 4.7 and 3000 μM.

### Culture of THP-1 cells

Human monocytic THP-1 cells (ATCC#TIB-202) were cultured in RPMI 1640 medium, supplemented with 10% heat-inactivated fetal bovine serum (FBS, Sigma-Aldrich), and 1% penicillin/streptomycin (Thermo Fisher Scientific). The cells were cultured in a humidified environment with 5% carbon dioxide (CO_2_) at 37 °C, and the culture medium was refreshed every 3 days. The cell density was kept within the range of 0.2–1.2 × 10^6^ cells/mL. THP‐1 monocytes were activated and polarized using established protocols [19]. Briefly, 5 × 10^5^/mL THP-1 monocytes were treated with 50 ng/mL phorbol myristate acetate (PMA, Sigma-Aldrich) for 48 h, facilitating the activation of monocytes into M0 macrophages.

### Influence of Zn^2+^ on macrophage viability

The metabolic activity of activated M0 macrophages was assessed utilizing cell counting kit 8 (CCK-8). A stock solution of 5 mM water-soluble tetrazolium 8 (WST-8, 5-(2,4- disulfophenyl)-3-(2-methoxy-4-nitrophenyl)-2-(4-nitrophenyl)-2H- tetrazolium, inner salt, monosodium salt, Cayman Chemicals) and 0.2 mM 1-methoxy-5-methylphenazinium (TCI Chemicals) was prepared in 150 mM sodium chloride (Merck) and stored at -80 °C until further use. THP-1 monocytes were added into a 48-well plate at a density of 5 × 10^5^/mL, and after activation they were subjected to treatment with 0.5 mL of Zn^2+^-containing media at concentrations from 4.7 to 3000 μM, with non-Zn^2+^-containing medium serving as the control group (n = 3). Following 24 h of incubation, cells were washed twice with Phosphate Buffered Saline (PBS), and then incubated with 500 μL of 10 v/v% CCK-8 solution in medium for 2 h. Subsequently, 100 μL of supernatant from each well was transferred to a 96-well plate, and the optical density (OD) value was spectrophotometrically measured using a microplate reader (Bio-Tek FL600 microplate fluorescence reader, Biotek) at a wavelength of 450 nm (OD_450_).

To assess macrophage viability, the dsDNA content of the M0 macrophages was quantified following a 24-h treatment with diverse Zn^2+^-containing media (n = 3). Subsequent to two PBS washes, 500 μL of Milli-Q was added to each well, and the samples underwent two cycles of freezing (− 20 °C) and thawing (room temperature). The quantification of dsDNA content was carried out using the QuantiFluor dsDNA Kit (Promega), adhering to the manufacturer's guidelines.

Simultaneously, LIVE/DEAD cell staining was conducted to visually evaluate cell viability. M0 macrophages were cultured on 8-well chamber slides (ibidi) at a density of 5 × 10^5^/mL. Based on the outcomes of the aforementioned measurements, Zn^2+^-containing media were refined to five concentrations: 4.7 μM, 37.5 μM, 300 μM, 600 μM, and 3000 μM. Following a 24-h incubation period, the cells underwent initial rinsing with Dulbecco's Phosphate-buffered saline (DPBS, Gibco) and subsequent exposure to a staining solution, prepared using the LIVE/DEAD™ Viability/Cytotoxicity Kit (Invitrogen™), as per the manufacturer's instructions. The stained cells were visualized using fluorescent microscopy (Axio Imager Microscope Z2, Zeiss).

### Influence of Zn^2+^ on macrophage polarization

Based on the cell viability assessments, for subsequent experiments the Zn^2+^ concentration range was narrowed down to 0–300 μM. Following activation, M0 macrophages were seeded onto 8-well chamber slides and subjected to treatment with culture media containing or lacking Zn^2+^. Control groups included the treatment of M0 macrophages for an additional 48 h with either 240 ng/mL LPS (Sigma-Aldrich) and 20 ng/mL INF-γ (Sigma-Aldrich) to induce M1 macrophages or 20 ng/mL IL-4 (Sigma-Aldrich) and 20 ng/mL IL-13 (Sigma-Aldrich) to induce M2 macrophages. After a 48-h of stimulation, cells were washed with PBS and fixed with 4% paraformaldehyde (PFA) for 10 min. Subsequent permeabilization with 0.5% Triton X-100 for 1 h at room temperature was followed by a 1-h blockage with 1% bovine serum albumin (BSA). The cells were then incubated overnight at 4 °C with rabbit anti-human CCR7 (1:250; abcam, No. ab32527) and mouse anti-human CD36 (1:100; Biolegend, No.336202) primary antibodies. The following day, after washing, incubation with Alexa Fluor 594-conjugated goat anti-rabbit and Alexa Fluor 488-conjugated goat anti-mouse secondary antibodies (both diluted at 1:200, Life Technologies) transpired for 1 h. Cell nuclei were stained by DAPI (1:2500, D9542, Sigma). Subsequently, stained cells were observed and imaged using a fluorescent microscope (Zeiss). Analysis for immunofluorescent images was performed through Image J (NIH). Macrophages were identified based on DAPI staining, and the M2/M1 macrophage ratio was calculated using CD36/CCR7-positive cells in two random fields per well. Each group comprised three wells.

### Cytokine secretion with Zn^2+^ stimulation

Following activation, M0 macrophages were seeded into a 6-well plate and subjected to treatment with or without Zn^2+^ (n = 3). Control groups included cells incubated with either IFN-γ/LPS or IL-4/IL-13. Post 48-h treatment, supernatant was collected from all wells for cytokine quantification. IL1-β, TNF-α and IL-10 levels were assessed utilizing a multiplex cytokine assay kit (Human Luminex® Discovery Assay, R&D Systems). TGF-β quantification was performed through an enzyme-linked immunosorbent assay (ELISA) kit (R&D Systems), adhering to the manufacturer's instructions.

### Zinc/Chitosan/Gelatin (Zn/CS/Gel) coating preparation and characterization

All chemicals required for the coating preparation were purchased from Sigma–Aldrich without further purification, except for those chemicals that were specifically mentioned. Commercially pure titanium (cpTi) disks, with a 15 mm diameter, were employed as substrates for the implant coatings. The coatings were deposited using the EPD technique. Prior to the deposition process, a zinc/chitosan (Zn/CS) chelate was prepared using the in-situ precipitation method by adding 7.85 g of ZnCl_2_·6H_2_O into 30 ml chitosan solution prepared by dissolving 0.6 g of chitosan (molecular weight ~ 190–310 kDa, DDA ~ 80%, Sigma) to a diluted acetic acid (Merk) solution (2% v/v) [20]. The same procedure was applied for the preparation of chitosan without zinc incorporation. The EPD suspension was prepared as reported previously [21]. In brief, Zn/CS chelate or pure chitosan (CS) were dissolved in a solvent mixture composed of acetic acid/water/ethanol (volume ratio = 1:20:79) with a pH of 4, reaching a concentration of 0.1 wt%. Simultaneously, a 0.2 wt% gelatin solution was prepared by dissolving type A gelatin (~ 179 kDa, kindly provided by Rousselot) into the same mixed solvent. The two solutions were combined in an equal volume ratio (1:1) after both powders were completely dissolved. The coatings were deposited using a direct current power supply (model 6614C; Agilent Technologies) at a constant voltage of 30 V based on our previous procedures [21]. After deposition, the coatings were rinsed with Milli-Q water to get rid of the undeposited suspension and air-dried at room temperature. Subsequently, the coatings were crosslinked using glutaraldehyde (25 wt% solution in water, ACROS Organics™) vapor at room temperature. A 0.1 M NaOH solution was used to neutralize any residual acid, whereafter the coated substrates were thoroughly washed with Milli-Q water to eliminate excess NaOH. The thickness of the coating was ~ 3 µm as measured by a profilometer (ProScan; Smithers). The Zn^2+^ release in PBS was measured under pH = 7 and 5.5 to mimic healthy and inflammatory microenvironments respectively. The released Zn^2+^, as measured by inductively coupled plasma atomic emission spectrometry (ICP-AES; 5100 SVDV, Agilent), was quantified as ~ 2.2 μg/mg coating at pH 7, and ~ 16.3 μg/mg coating at pH 5.5 after 3 days, and ~ 3 μg/mg coating at pH 7, and ~ 22.5 μg/mg coating at pH 5.5 after 2 weeks [22].

### Macrophage adhesion and morphology on coatings

All specimens were placed in 24-well plates and subjected to sterilization using argon plasma treatment for 3 min, facilitated by a radiofrequency glow discharge plasma cleaner (PDC-001; Harrick Scientific Corp.) [21]. Macrophages were cultured on various surfaces for 1 and 3 days. At each time point, samples were washed with PBS twice, and subsequently immersed in 1 mL of Milli-Q. Following the freezing–thawing cycles, the quantification of dsDNA content was performed using the QuantiFluor dsDNA Kit following the previously described procedure in Sect. 2.3 (n = 3).

To observe cell morphology, following a 3-day culture of macrophages on distinct surfaces, cells underwent PBS washing and fixation in a 2 wt% glutaraldehyde solution in 0.1 M sodium-cacodylate for 20 min. Subsequently, cells on the substrates were dehydrated using a series of graded ethanol concentrations (ranging from 50 to 100% ethanol, alongside 100% water-free ethanol) for 5 min each. Post-dehydration, the samples were air-dried in tetramethylsilane. The samples were sputter-coated with gold prior to observation under a scanning electron microscope (SEM, Sigma-300, Zeiss).

### Macrophage viability on coatings

Macrophages were seeded onto various surfaces at a density of 5 × 10^5^/mL. For the preparation of control groups, THP-1-derived macrophages were cultured on cpTi surfaces for M0, with M1 achieved through IFN-γ and LPS stimulation, and M2 through IL-4 and IL-13 stimulation [19]. Following a 24-h incubation period, LIVE/DEAD cell staining was executed to assess the cytotoxicity of the coatings to macrophages as described above. Simultaneously, the metabolic activity of macrophages on various surfaces was measured using CCK-8, following the previously outlined protocol. Each experimental group underwent testing with three samples (n = 3).

### Macrophage polarization on coatings

After culturing THP-1 derived macrophages on different surfaces for 3 days, the supernatant was collected for cytokine quantification. Subsequently, all samples were washed with PBS and fixed with 4% PFA. Staining procedures were consistent with those aforementioned in Sect. 2.4. Following staining, samples were mounted with ProLong™ Gold Antifade Mountant (Invitrogen™). The stained samples were subsequently observed and imaged using scanning fluorescent laser microscopy (TCS SP8 SMD, Leica). Analysis for immunofluorescent images was carried out using Image J (NIH). Three samples were involved in each group, and 2 images were randomly taken from each sample.

The supernatant obtained from macrophage cultures on various surfaces were centrifuged at 400 g for 10 min. Subsequently, the levels of IL-1β, IL-6, TNF-α and IL-10 were assessed utilizing a multiplex cytokine assay kit (Human Luminex® Discovery Assay, R&D Systems). TGF-β was quantified using an ELISA assay kit. Three samples were included in each group.

#### Preparation of conditioned media and hGFs culture

For preparation of conditioned media (CM), THP-1 derived macrophages were cultured on different substrates for 3 days. The media were collected and centrifuged at 400 g for 10 min. The supernatants were mixed with fibroblast culture medium at a ratio of 1:1 and kept at -80 °C for subsequent experiments.

Human gingival fibroblasts (hGFs), obtained from healthy human gingival tissues, were cultured in Dulbecco's modified Eagle's medium (DMEM; Gibco) with 10 vol% FBS; and 1% penicillin/streptomycin. The hGFs were cultured in a humidified environment containing 5% CO_2_ at 37 °C, and the culture medium was refreshed every 3 days. hGFs were cultured on either µ-Slide 8 Well chamber slides (ibidi) or 96-well plates with a density of 0.75 × 10^4^/cm^2^. After overnight attachment, fibroblast culture medium were replaced by CM for further treatment. For convenience, the group of hGFs cultured in different CM were denominated as followes: CM-M1 (CM derived from cpTi-LPS/IFN-γ group), CM-M2 (CM derived from cpTi-IL-4/IL-13 group), CM-M0 (CM derived from cpTi group), CM-CS/Gel (CM derived from CS/Gel group), CM-Zn/CS/Gel (CM derived from Zn/CS/Gel group).

#### Responses of hGFs in conditioned media

After exposure to CM for 24 h, LIVE/DEAD cell staining was carried out to assess hGF viability on µ-Slide 8 Well chamber slides. The staining process was as same as described above in Sect. 2.4. Cell metabolic activity was evaluated as a function of various CM stimulations using the CCK-8 assay after incubation for 1, 3, and 7 days. At each time point, hGFs cultured on 96-well plates were subjected to two PBS washes and then incubated with 100 μL of 10 v/v% CCK-8 solution in medium for 2 h. Subsequently, the OD value was spectrophotometrically measured. Each group was tested with four wells.

To access cell adhesion, following the 24-h stimulation with CM, fibroblasts were stained by vinculin antibody (diluted at 1:100, ab129002, Abcam), F-actin (diluted at 1:200, #A-12379, Invitrogen) and DAPI (1:2500, D9542, Sigma) to access their adhesion. Stained samples were observed and imaged using fluorescent microscopy (Leica) (n = 4).

To evaluate the collagen deposition by hGFs under different immune microenvironment, cells were cultured with different CM for one week. Subsequently, the cells were stained with Col I antibody (diluted at 1:100, ab233080, Abcam), F-actin and DAPI, and then observed using a fluorescent microscope (Leica) (n = 4). The mean fluorescence intensity was calculated through ImageJ (NIH) by measuring the fluorescence intensity and area based on a reported protocol [23].

To further quantify mitochondrial activity, hGFs were treated by different CM for 24 h (n = 4). Subsequently, the cells were washed with PBS and incubated with the MitoTracker® probe (Invitrogen) and Hoechst (Thermo Scientific™) at 37 °C for 30 min following the manufacturer’s guidelines. Subsequently, the incubated cells were observed and imaged using a fluorescent microscope (Leica). Mitochondrial parameters including number, area and branch number were measured based on binary images in ImageJ by using the Mitochondria Analyzer plugin according to previous study [24].

### Statistical analysis

Statistical analyses were conducted using GraphPad Prism software. Data are presented as mean ± standard deviation (SD), with sample size information explicitly detailed in the experimental methods section. Aspects of cellular performance were statistically analyzed using one-way ANOVA with Tukey’s post-hoc test for multiple group comparisons. A significance threshold of *p* < 0.05 was considered.

## Results and discussion

### Effect of Zn^2+^ on macrophage viability

Only a few studies have employed THP-1 derived macrophages to assess the immunomodulatory function of zinc in tissue regeneration [25]. In addition, the therapeutically effective concentrations of zinc are not clear. Hence, this study explored the effect of zinc on THP-1 derived macrophages by incubating cells in culture media with different Zn^2+^ concentrations.

**Fig. 1:**
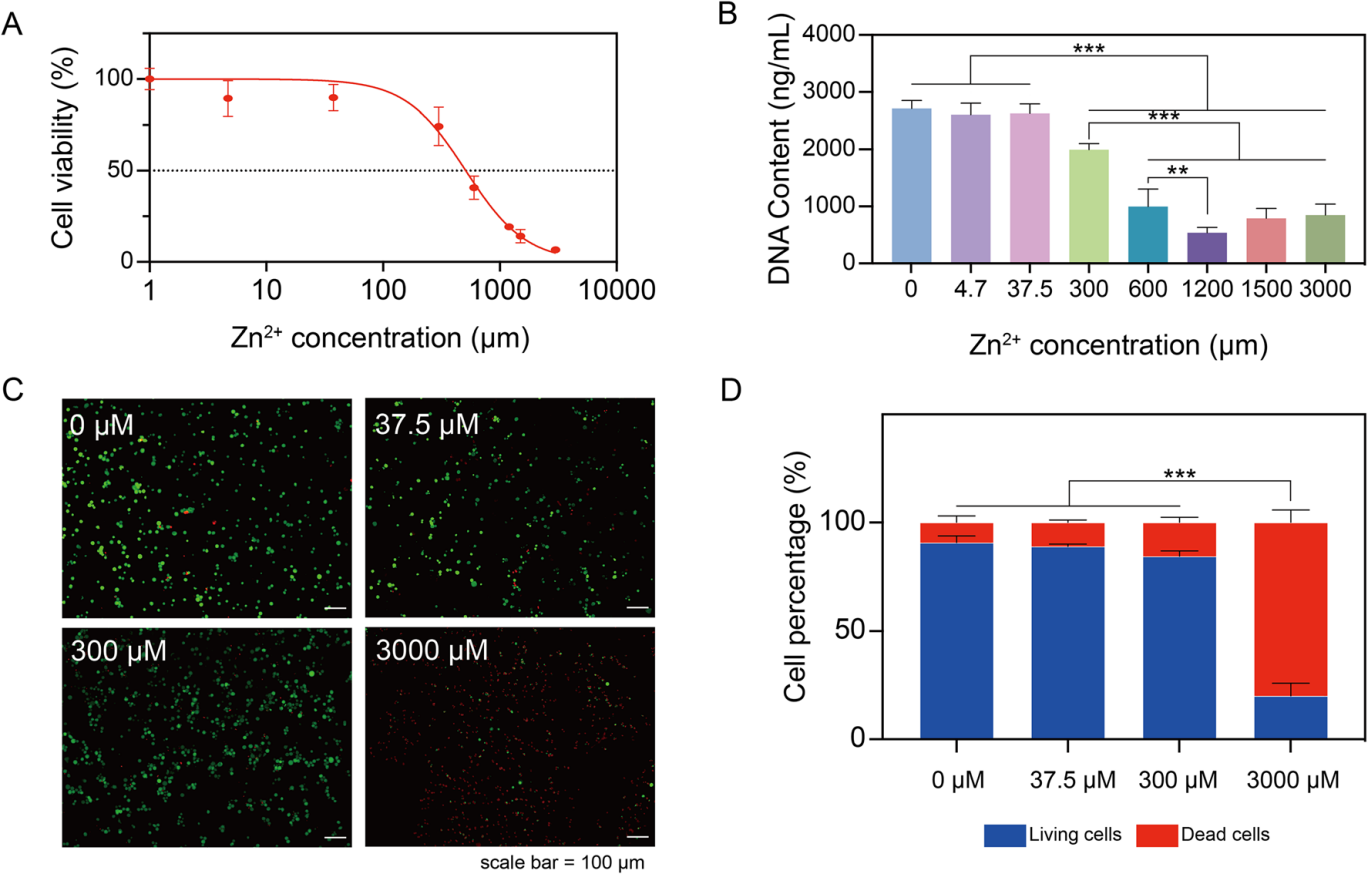
Zn^2+^ effects on macrophage viability. **A** THP-1 derived macrophage viability in percentage (%) after 24-h exposure to Zn^2+^ containing media. **B** dsDNA content of cells after 24-h incubation with different concentrations of Zn^2+^. **C** Fluorescent images of living (green) and dead cells (red) after incubation with Zn^2+^ containing media for 24 h; scale bar = 100 μm. **D** Quantification of living and dead cells depicted in total cell percentage (%). These findings revealed a dose-dependent toxic effect of Zn.^2+^ on THP-1 derived macrophages. **p < 0.01, ***p < 0.001

After a 24-h incubation period, the metabolic activity of the macrophages was determined. Subsequently, the half maximal inhibitory concentration (IC_50_) was determined to characterize the cytotoxic inhibitory potency of Zn^2+^ on macrophages. Results indicated that lower concentrations of Zn^2+^ (4.7 and 37.5 μM) did not significantly affect macrophage viability, while a gradual decline in macrophage cell viability was observed upon exposure to Zn^2+^  ≥ 300 μM, with an IC_50_ of 510 μM. Further increase of Zn^2+^ concentrations (3000 μM) resulted in diminished cell metabolism and severely affected cell viability (Fig. 1A).

Cell numbers were assessed through dsDNA content quantification after exposing THP-1 derived macrophages to different concentrations of Zn^2+^. The results revealed a trend which was consistent with the above-described viability measurements (Fig. 1B). A decline in dsDNA content was observed for treatments ≥ 300 μM Zn^2+^ (*p* < 0.001). Higher Zn^2+^ concentration from 600 μM to 1200 μM resulted in a significant reduction of the dsDNA content (*p* < 0.001), and above 1200 μM Zn^2+^ the dsDNA content of macrophages remained consistently low, showing no statistically significant difference compared to the groups at 600 μM and 1200 μM.

Cell viability was further assessed using a LIVE/DEAD staining assay (Fig. 1C). Considering the results from the CCK-8 and dsDNA assays, we excluded treatments with 1200 μM and 1500 μM Zn^2+^. Consistent with previous observations, LIVE/DEAD staining revealed an increase in dead cells (red) with a decrease in living cells (green) as the Zn^2+^ concentration increased from 300 μM (Fig. 1C). Quantification of the LIVE/DEAD results indicated a clear reduction of viable cells at a threshold concentration between 300 and 3000 μM (*p* < 0.001), which was in line with cell viability and dsDNA content measurements (Fig. 1D).

Together, these findings revealed a dose-dependent toxic effect of Zn^2+^ on THP-1 derived macrophages. Previous studies have reported various safety concentration ranges for Zn^2+^. For instance, Song et al. reported an IC_50_ of ZnCl_2_ at 205.1 μM in mouse Ana-1 macrophages [26], while Yoshikawa et al. reported an IC_50_ of ZnSO_4_ in rat adipocytes at ~ 1580 μM [27]. The safety concentrations of Zn^2+^ for human macrophages observed in the current study are in agreement with these reported concentration ranges. However, the reported concentration ranges were different for various studies, most likely due to different cell models and experimental setups. Previous work has attributed Zn^2+^ cytotoxicity to the formation of zinc-containing nanoparticles in cell culture media at a range between 50 to 30,000 μM [28]. However, we did not observe similar flocculent white precipitates in any Zn^2+^-supplemented cell culture media. Consequently, based on our cell viability results, we narrowed the Zn^2+^ concentration range to 0–300 μM in subsequent studies.

### Effect of Zn^2+^ on macrophage polarization

**Fig. 2:**
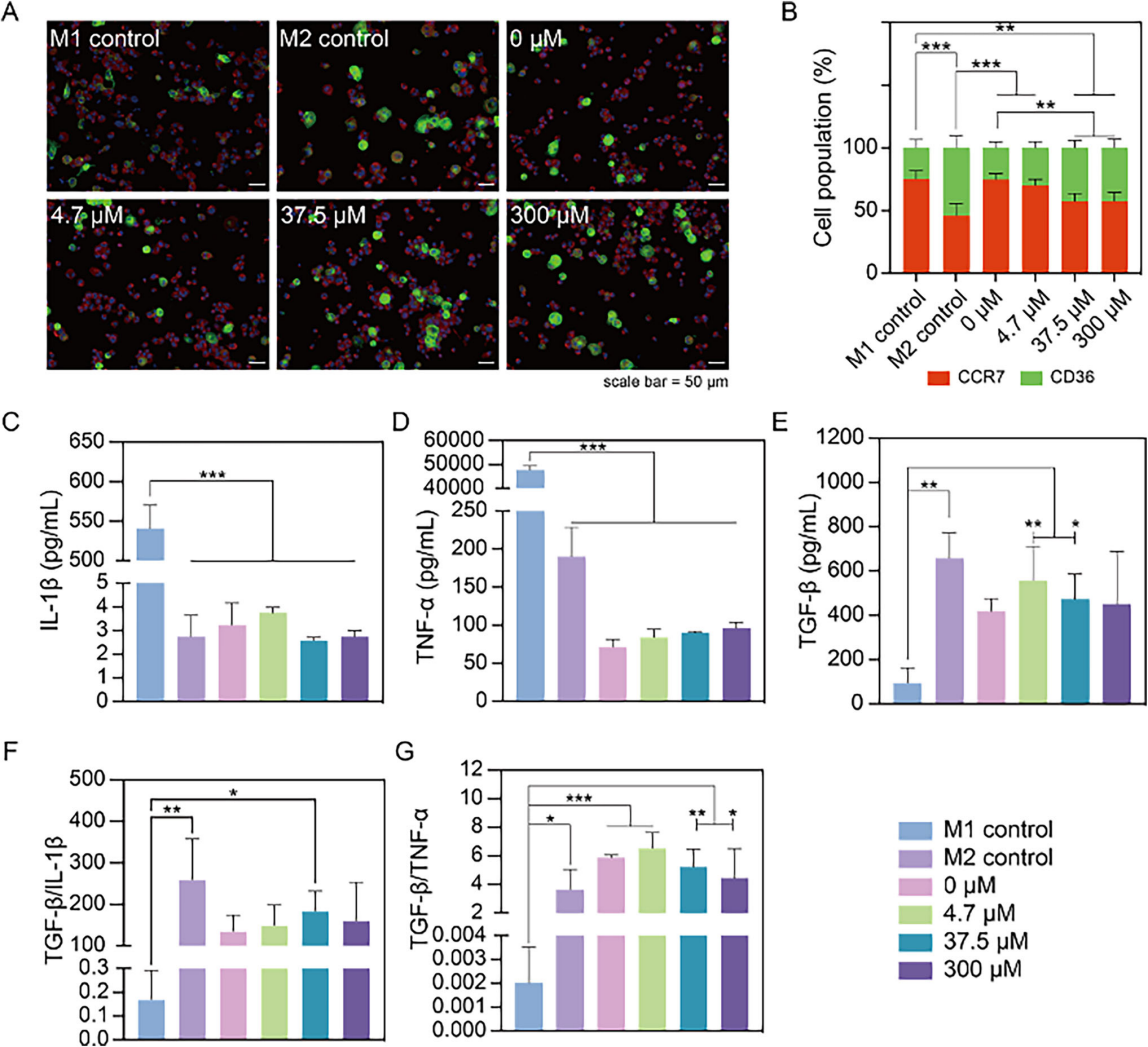
Phenotypic characterization and secretome analysis of macrophages upon 48-h culture in Zn^2+^ containing media. **A** Immunostaining images of macrophages with M1 macrophage marker CCR7 (red) and M2 macrophage marker CD36 (green); scale bar = 50 μm. **B** Quantification of CCR7 and CD36 positively stained cells from fluorescent microscopy, depicted as total cell population percentage (%). **C** Secretion of pro-inflammatory cytokines IL-1β, **D** TNF-α; and **E** anti-inflammatory cytokine TGF-β. **F** Ratio of TGF-β and IL-1β, and **G** TGF-β and TNF-α. Results indicate that exposure of M0 macrophages to mild Zn.^2+^ concentrations favors macrophage polarization toward the M2 phenotype. *p < 0.05, **p < 0.01, ***p < 0.001

To assess the impact of Zn^2+^ on macrophage polarization, THP-1 derived macrophages were exposed to Zn^2+^-containing media. Control groups contained M0 macrophages that were polarized toward either the M1 or M2 phenotype using LPS/IFN-γ or IL-4/IL-13, based on an established protocol [19]. Following a 48-h incubation, the specific macrophage phenotype was determined as a function of Zn^2+^ concentration by staining with the M1 macrophage surface marker CCR7 (red) and the M2 macrophage surface marker CD36 (green). Both control groups treated with either LPS/IFN-γ or IL-4/IL-13 exhibited CCR7 and CD36 expression, corresponding to non-exclusive marker expression for M1 and M2 human macrophages. However, cells treated with LPS/IFN-γ expressed CCR7 in a more pronounced manner, while cells treated with IL-4/IL-13 showed higher CD36 expression (Fig. 2A), consistent with a previous study from our group [19]. Quantitative analysis of the images revealed that macrophages treated with LPS/IFN-γ exhibited a significant increase in CCR7 staining (75.1% ± 6.6%) compared to cells treated with IL-4/IL-13 (46.1% ± 9.5%; *p* < 0.001). Conversely, macrophages treated with IL-4/IL-13 contained a higher proportion of CD36-positive cells (53.9% ± 9.5%) than cells treated with LPS/IFN-γ (24.9% ± 6.6%; *p* < 0.001) (Fig. 2B). Without exposure to Zn^2+^, macrophages tended toward an M1 phenotype. With increasing Zn^2+^ concentrations, the proportion of CD36-positive cells increased, indicating a tendency toward an M2 phenotype. Specifically, at Zn^2+^ concentrations of 37.5 μM, the population of CD36-positive cells showed a 1.5-fold increase compared to 0 μM group (*p* < 0.01). An additional increase of the Zn^2+^ concentration to 300 μM did not enhance CD36-staining.

To further elucidate the effect of Zn^2+^ on macrophage polarization, we assessed the secretion of pro-inflammatory cytokines IL-1β and TNF-α, as well as pro-regenerative cytokines IL-10 (data not shown as all values were below the detectable standard range) and TGF-β (Fig. 2C-E). Our results revealed that IL-1β and TNF-α were significantly elevated for M1 controls (*p* < 0.001). Treatments with different Zn^2+^ concentrations did not affect the secretion of cytokines compared to either 0 μM Zn^2+^ or M2 controls. To provide a comprehensive assessment of the most crucial pro- and anti-inflammatory cytokine secretion, we calculated the ratio of anti- to pro-inflammatory cytokines (TGF-β/IL-1β and TGF-β/TNF-α) (Fig. 2F, G). M1 controls exhibited the lowest ratio of anti- to pro-inflammatory cytokines compared to other groups (*p* < 0.05), while macrophages exposed to Zn^2+^ showed similar cytokine secretion levels as compared to M2 controls.

Based on the above-described results we conclude that exposure of M0 macrophages to mild Zn^2+^ concentrations favors macrophage polarization toward the M2 phenotype. Therefore, we developed novel Zn-containing coatings and studied their immunomodulatory effects from the perspective of potential application in peri-implant wound healing.

### Coating characteristics

Chitosan is a biocompatible polymer, which has been commonly used for biomedical applications and for electrophoretic deposition (EPD) coatings. The cytocompatibility of chitosan EPD coatings for dental application was confirmed in our previous publication [21]. However, our results also indicated that chitosan alone did not sufficiently support cell adhesion. To address this, we incorporated gelatin into our coatings in the subsequent studies, as the RGD (Arg- Gly-Asp) sequences in gelatin are known to enhance cell adhesion [22].

Detailed characterization of the coatings by using scanning electron microscopy, energy-dispersive X-ray spectroscopy techniques, water contact angles, adhesion strength, Fourier-transform infrared spectroscopy and zinc release profile can be found in our previous publication [22]. The coatings remained stable, showing no morphological changes up to the maximum experimental period, i.e. > 7 days. The swelling and biodegradability of the coating were not tested in the current study. A former study reported that the swelling rate of a chitosan/gelatin scaffold was around 80% and 290% after 12 h and 24 h, respectively, in PBS [29]. Additionally, it was observed that the chitosan–gelatin matrix could maintain its microstructure for up to 8 weeks, with chitosan remaining present. These findings suggest that the physical properties of the coatings may align well with the healing process of peri-implant gingival tissue [30].

The release of Zn^2+^ can be regulated by the amount of zinc incorporated and the pH, as demonstrated in our previous article [22]. In the current Zn/CS/Gel coating, where the highest zinc content was incorporated, a sustained release of Zn^2+^ was achieved at approximately 4.62 μM under pH = 7 and 34.62 μM under pH = 5.5. Both concentrations were below the cytotoxic threshold, as confirmed by the results of this study (Fig. 1).

### Effects of Zn/CS/Gel coatings on macrophage behavior

To explore the immunomodulatory effects of Zn/CS/Gel coatings, THP-1 derived macrophages were cultured on both experimental coatings and uncoated cpTi control surfaces. To ensure comparability, we established three control conditions, wherein macrophages were cultured on uncoated cpTi surfaces upon stimulation of polarization into either LPS/IFN-γ stimulated M1 or IL-4/IL-13 stimulated M2 macrophages. We assessed *in vitro* cell adhesion, cytocompatibility, and polarization state.

**Fig. 3:**
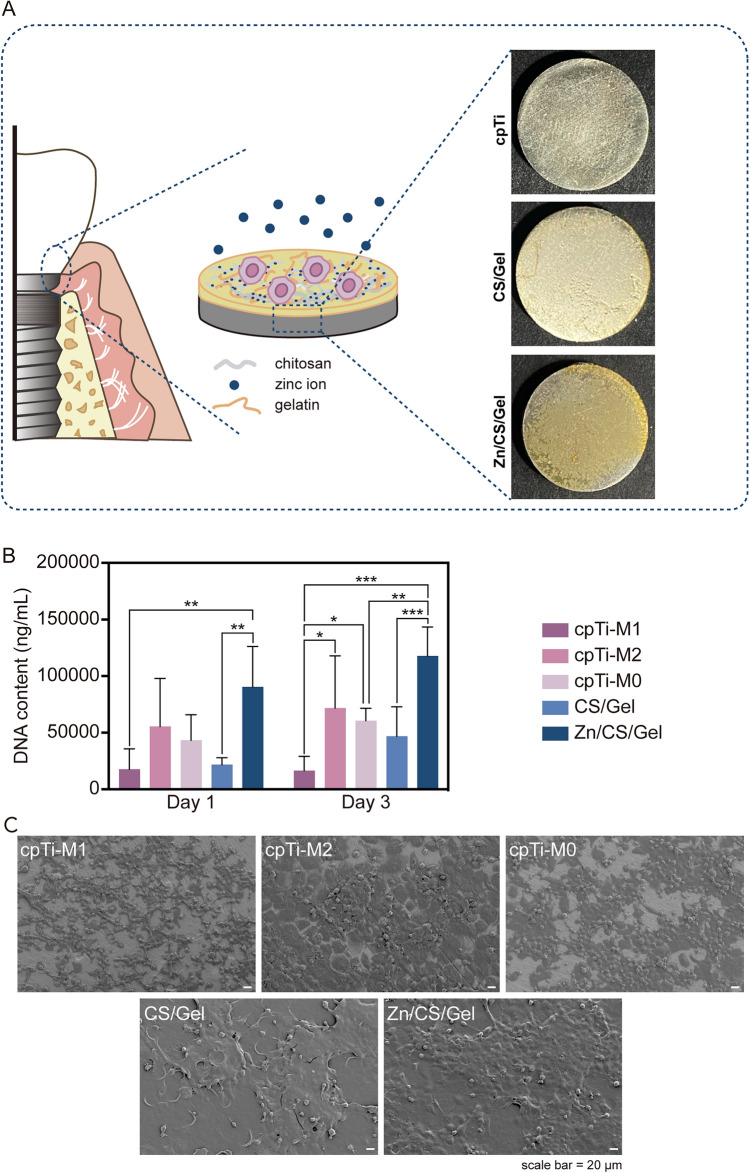
Effects of Zn/CS/Gel coatings on macrophage adhesion. **A** Schematic illustration of cell seeding and digital photographs of the substrates; **B** dsDNA content of macrophages after culture on different surfaces for 1 and 3 days; **C** SEM images of macrophages on different surfaces after 3 days of culture; scale bar = 20 μm. Results indicate that Zn.^2+^ stimulated macrophage adhesion to this polymer-based surface. *p < 0.05, **p < 0.01, ***p < 0.001

To investigate the adhesion of THP-1 derived macrophages on different surfaces, the amount of dsDNA of adhered cells was quantified. For cells cultured on cpTi, the adhesion of M1 macrophage controls was significantly lower than the other two controls, particularly after 3 days of culture (*p* < 0.05). Macrophages in the Zn/CS/Gel group exhibited superior adhesion compared to all other groups, either at Day 1 or Day 3. Specifically, compared to macrophages cultured on cpTi surfaces without extra stimulation, cells cultured on coatings (w/- Zn) showed distinct adhesion characteristics. The CS/Gel coating reduced macrophage adhesion, while Zn/CS/Gel significantly enhanced macrophage adhesion (*p* < 0.01) (Fig. 3A), suggesting that Zn^2+^ stimulated macrophage adhesion to this polymer-based surface. The absence of a similar phenomenon in the previous study involving Zn^2+^ treatment might be attributed to the initiation of macrophage stimulation after their differentiation from THP-1 monocytes in tissue culture plates. When we cultured macrophages on disks, we directly added the THP-1 monocyte suspension to the wells containing different substrates. Moreover, prior studies have reported that macrophage adhesion to titanium surfaces is mainly dependent on chemical properties rather than surface roughness, which aligns with our observations [31, 32]. Additionally, the observed improvement in macrophage adhesion on Zn^2+^-containing coatings compared to zinc-free surfaces is consistent with findings from previous studies [33, 34].

SEM images revealed different macrophage morphologies for the various experimental groups. Macrophages adhered abundantly on both coating types, especially on Zn/CS/Gel coatings (Fig. 3B). Previous studies reported that anti-inflammatory cytokines like TGF-β regulate the expression of cell adhesion molecules, such as integrins, and hence affect the adhesion of macrophages [35, 36]. Additionally, TGF-β can also control intracellular signaling pathways, such as the SMAD signaling pathway, thereby influencing gene expression related to macrophage adhesion and the reorganization of the cellular cytoskeleton [37, 38].

**Fig. 4:**
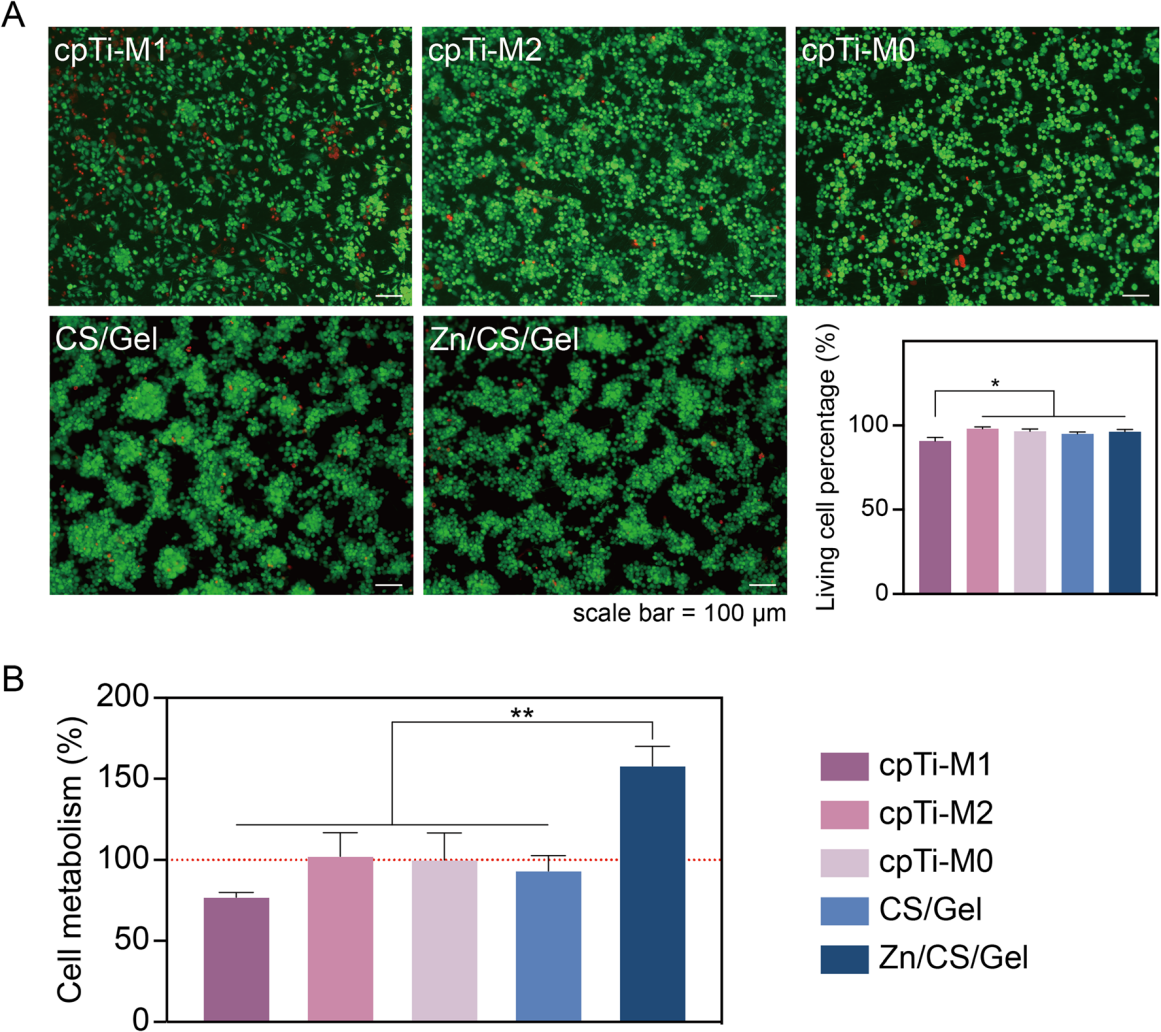
Effects of coatings on THP-1 derived macrophages viability. **A** LIVE/DEAD cell staining after culturing macrophages for 24 h; scale bar = 100 μm. **B** Quantification of the percentage of live cells, depicted as total cell population percentage (%); **C** macrophage viability assessed using the CCK-8 assay after 24 h of cell culture (cell metabolism in cpTi-M0 was regarded as 100% indicated by red dashed line). Results indicate that Zn.^2+^-containing coating enhances cellular metabolic activity of macrophages. *p < 0.05, **p < 0.01

To determine coating cytocompatibility, THP-1 derived macrophages were seeded onto various surfaces. Observations from the microscopy images revealed that the majority of macrophages were alive (green fluorescent staining), suggesting cytocompatibility of all surfaces for THP-1 derived macrophages, except for an increased number of dead cells (red fluorescent staining) upon treatment with LPS/IFN-γ. Compared to cells on pure titanium, macrophages on the coatings tended to cluster, which corresponds to our SEM observations (Fig. 4A). Quantification of the percentage of live cells confirmed our observations, indicating slightly lower viability of macrophages for cpTi-M1 controls compared to other groups. This decrease may be attributed to the inflammatory status induced by pro-inflammatory factors, leading to reduced cell viability [39].

Furthermore, cell metabolism was measured through a CCK-8 assay after culturing THP-1 derived macrophages for 24 h. Interestingly, cells on Zn/CS/Gel coatings exhibited the highest metabolic activity compared to the cells in other groups (*p* < 0.01) (Fig. 4B). Considering that THP-1 derived macrophages do not proliferate once activated, we hypothesize that the Zn^2+^-containing coating may enhance cellular metabolic activity. Combining these results with our previous study where THP-1 derived macrophages were incubated with Zn^2+^ containing media, we speculate that the increased metabolism observed Zn/CS/Gel coatings is likely due to enhanced cell adhesion to this surface.

**Fig. 5:**
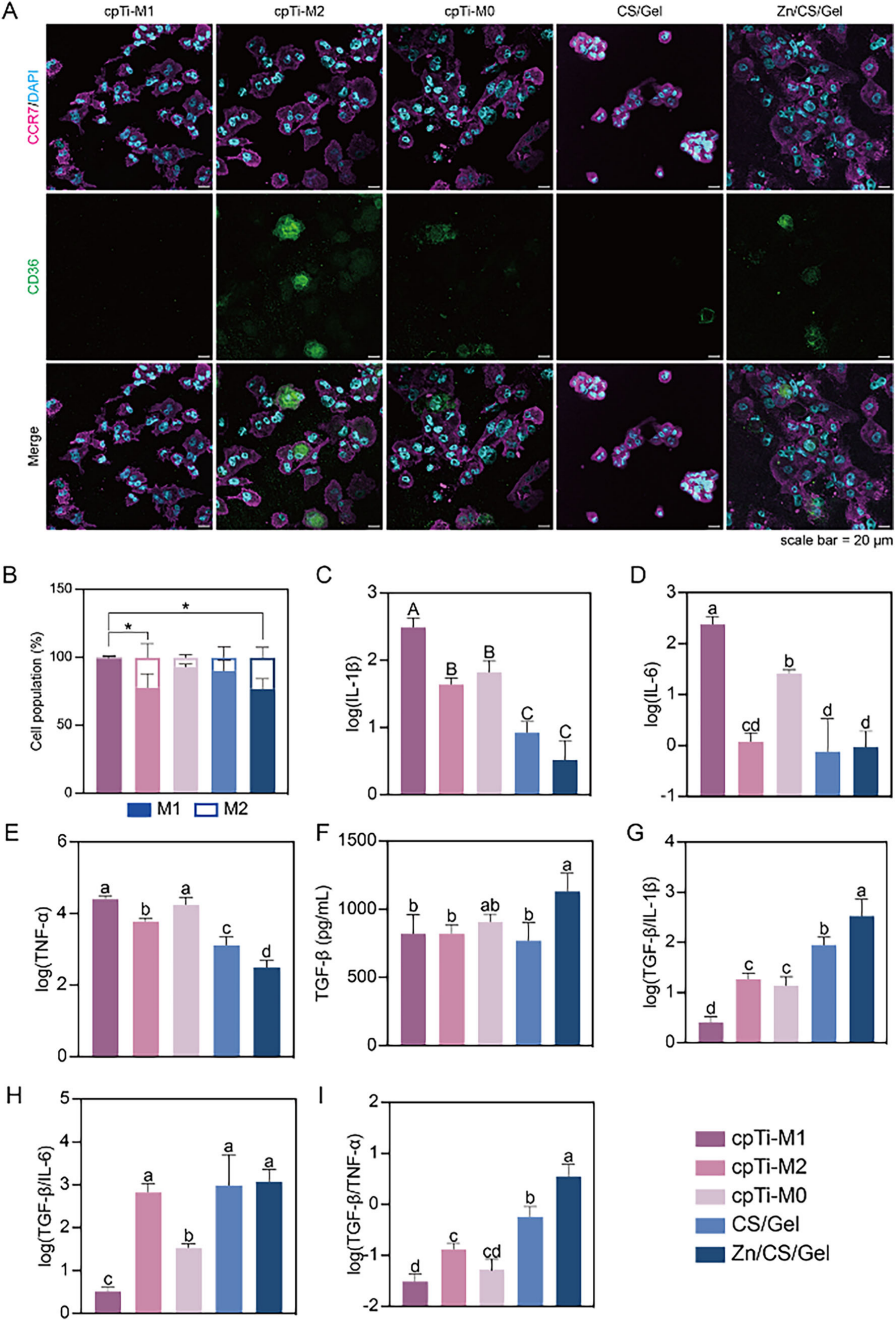
Effects of Zn/CS/Gel coatings on macrophage polarization and secretome upon culture of 3 days. **A** Immunostaining images of macrophages with the M1 macrophage marker CCR7 (magenta) and the M2 macrophage marker CD36 (green); scale bar = 20 μm. **B** Quantification of M1 and M2 macrophage population, depicted as total cell population percentage (%). **C** IL-1β, **D** IL-6; **E** TNF-α, and anti-inflammation cytokines **F** TGF-β. Ratio of (**G**) TGF-β/IL-1β, **H** TGF-β/IL-6; **I** TGF-β/ TNF-α. These findings demonstrate that Zn/CS/Gel coatings modulate the inflammatory response by downregulating the pro-inflammatory cytokine secretion and upregulating anti-inflammatory cytokine secretion. *p < 0.05. a: significance level = 0.05; A: significance level = 0.01

To ascertain the polarization of macrophages cultured for 3 days on different surfaces, immunofluorescent staining was employed to label CCR7 (magenta, M1 macrophage marker) and CD36 (green, M2 macrophage marker) in THP-1 derived macrophages. Macrophages treated with LPS/IFN-γ as control exhibited substantial CCR7 expression with limited CD36 expression, while the other two controls displayed both CCR7 and CD36 expression. Macrophages cultured on both types of coating similarly exhibited both CCR7 and CD36 expression (Fig. 5A).

Quantitative analysis of macrophage polarization (Fig. 5B) indicated less CCR7 staining for macrophages on Zn/CS/Gel coatings compared to M1 control macrophages (*p* < 0.05). Moreover, macrophages cultured on Zn/CS/Gel coatings exhibited comparable CD36 expression to M2 control macrophages on cpTi surfaces. Additionally, macrophage morphology correlated with the earlier SEM observations indicating that M1 macrophages were smallest, while macrophages cultured on Zn/CS/Gel coatings showed enhanced spreading (Figs. 3B and 5A).

Our results revealed a higher amount of M2 macrophages on Zn/CS/Gel coatings, which aligns with findings from previous studies [33, 34]. While various surfaces induced macrophage polarization to different extents, none of the groups in these previously performed studies distinctly directed THP-1 derived macrophages into the M1 or M2 phenotypes. Herein, on the contrary, we employed M1 and M2 controls stimulated according to an established protocol on titanium surfaces. Previous studies have emphasized that material-activated macrophage phenotypes may not precisely mirror conventionally cytokine-activated states due to the intricate nature of macrophages [19]. A decrease in M1 phenotype characteristics does not necessarily correlate with a simultaneous increase in M2 phenotype characteristics and vice versa. A key factor of macrophage-mediated immunomodulatory functions in wound healing involves cytokine secretion. Therefore, for assessment of immunomodulatory response of macrophages on biomaterials, measurement of cytokine secretion levels and quantification of M1 and M2 morphological phenotypes are essential.

To further validate macrophage polarization on different surfaces, we assessed the secretion of representative pro- and anti-inflammatory cytokines and using M1 and M2 macrophage controls. Results revealed that M1 control macrophages exhibited the highest secretion of IL-1β and IL-6, while this secretion of inflammatory mediators significantly decreased for M2 control macrophages, compared to the other two controls (*p* < 0.05) (Fig. 5C, D). Macrophages cultured on either type of coating displayed reduced secretion of inflammatory mediators, particularly those cultured on Zn/CS/Gel coatings (Fig. 5C-E). Similar to our former measurements, the concentration of IL-10 was below the limit of detection. TGF-β secretion was highest for macrophages cultured on Zn/CS/Gel coatings (*p* < 0.05) (Fig. 5F). The ratio of anti- to pro-inflammatory cytokines (TGF-β/IL-1β, TGF-β/IL-6, and TGF-β/TNF-α) showed to be highest for macrophages cultured on Zn/CS/Gel coatings (Fig. 5G-I).

These findings demonstrate that Zn/CS/Gel coatings effectively modulate the inflammatory response by downregulating the secretion of pro-inflammatory cytokines and upregulating the secretion of anti-inflammatory cytokines. The ability of zinc-based materials to facilitate the M1-to-M2 macrophage transition and promote wound healing has been well documented [17]. Hereby, we provided a new strategy to load and release Zn^2+^ through a simple and cost-effective coating deposition technique. In addition, effective and timely modulation of M1 and M2 macrophage polarization, along with the induction of cytokine secretion, is crucial to improve the outcome of wound healing. Yu et al. demonstrated that zinc-coated scaffolds increased M1 polarization within the initial 6 h, followed by a subsequent increase in M2 response after 24 h [40]. Bai et al. reported that zinc-doped porous microcrystalline bioactive glass facilitates an M1-to-M2 transition within 3–7 days [41]. Additionally, Lu et al. have shown that macrophages cultured on calcium phosphate cement incorporated with zinc silicate undergo M1-to-M2 conversion by day 3 [42]. These findings align with our observations after culturing macrophages on Zn/CS/Gel coatings for 3 days. Hence, we postulate that Zn/CS/Gel coatings exert immunomodulatory capacity by favoring macrophage polarization toward the M2 type.

### Indirect effects of Zn/CS/Gel coatings on fibroblast behavior via macrophage-conditioned media

**Fig. 6:**
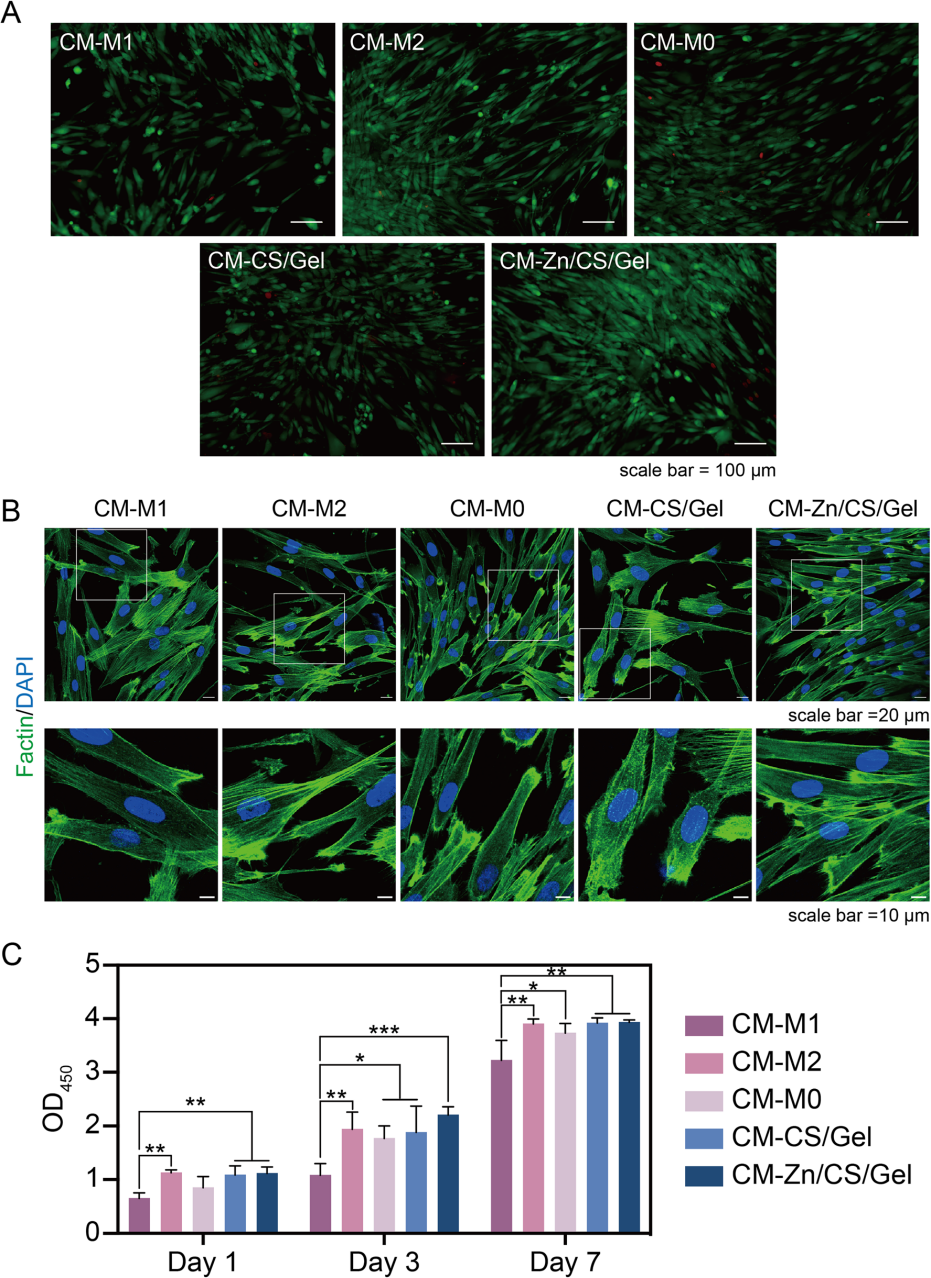
Indirect effects of Zn/CS/Gel coatings on hGF viability. **A** LIVE/DEAD cell staining after 24-h incubation; scale bar = 100 μm. **B** hGFs morphology upon culture in CM after cytoskeleton staining; upper image scale bar = 20 μm, lower image scale bar = 10 μm. **C** hGF viability after incubation with CM for 1, 3 and 7 days. Results showed that CM-M1 compromised fibroblast growth. *p < 0.05, ** p < 0.01, *** p < 0.001

Since the immune environment strongly determines tissue integration, we subsequently investigated the implication of the macrophage-derived immune microenvironment on cellular behavior of soft tissue cells. Hereby, we used hGFs as model cells, since they are dominant within peri-implant soft tissue, functioning as important cellular components for the formation of a soft tissue seal [7]. Before studying the effects of differential immune stimulation on fibroblast cellular function, we verified fibroblast viability under CM stimulation obtained from macrophage cultures. After 24-h incubation, LIVE/DEAD staining indicated that cell viability was not compromised under any of the simulated immune environments (Fig. 6A). Fibroblast cell morphology showed a typical elongated spindle shape without apparent differences (Fig. 6B). Results of CCK-8 viability assays showed that fibroblasts generally grew well, as reflected by continuous proliferation up to 7 days; lowest cell viability was observed for fibroblasts cultured in CM-M1 (*p* < 0.05) (Fig. 6C).

**Fig. 7:**
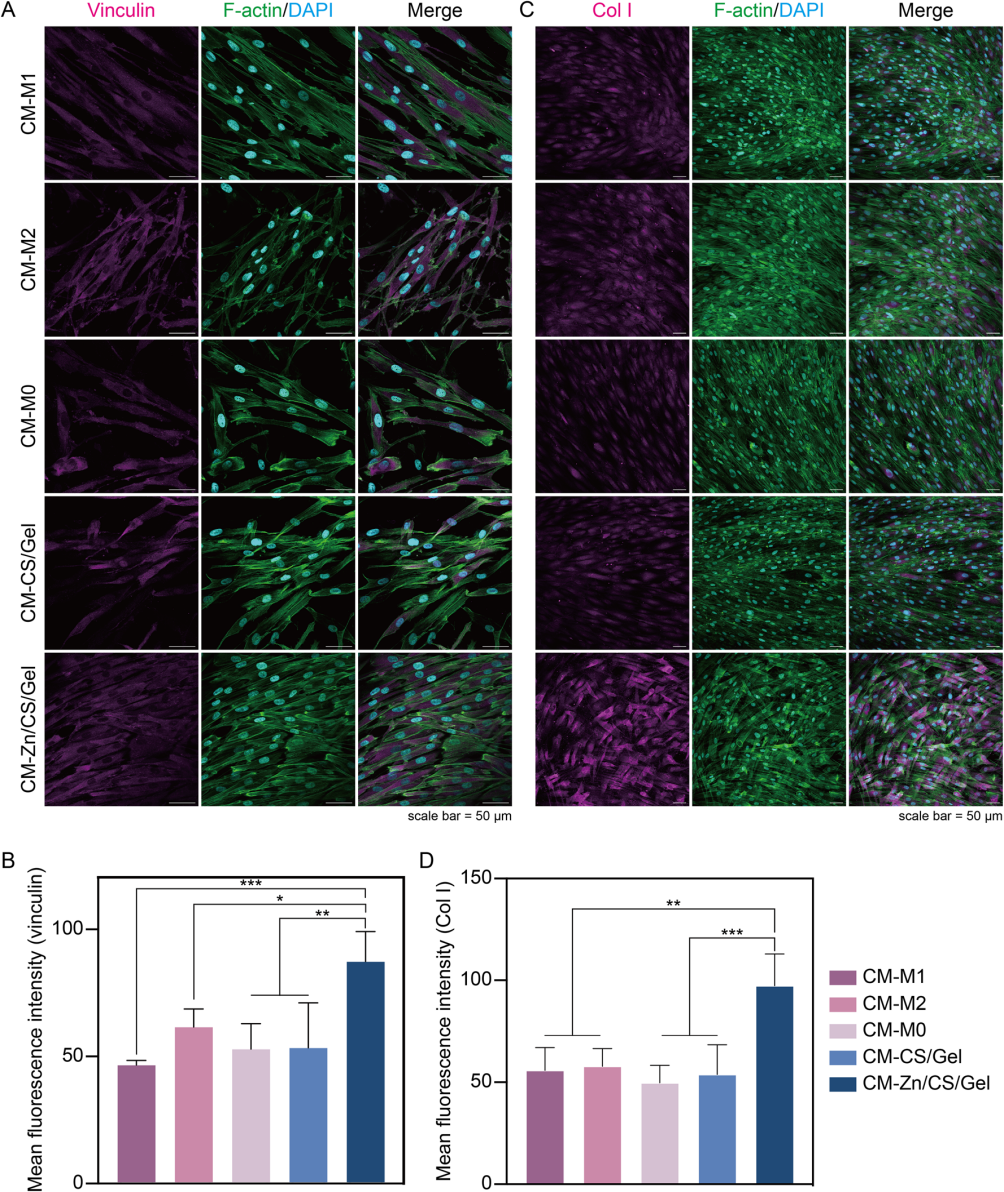
Indirect effects of Zn/CS/Gel coatings on hGF behavior. **A** Immunofluorescence staining of vinculin upon 1-day culture; scale bar = 50 μm. **B** Quantitative vinculin staining; **C** Immunofluorescence staining of Col I upon 7-day culture; scale bar = 50 μm. **D** Quantitative Col I staining. Results showed that CM-Zn/CS/Ge could create a pro-regenerative microenvironment beneficial for fibroblast adhesion and collagen deposition. *p < 0.05, ** p < 0.01, *** p < 0.001

To establish a robust peri-implant soft tissue seal, one of the most vital prerequisites is to achieve effective cell adhesion for soft tissue cells. Fibroblasts adhere to implant surfaces by developing focal adhesions, which are structures connecting the cytoskeleton, cell adhesive receptors, and ECM to an implant surface [43]. Vinculin is a cytoskeleton-related protein, which is a representative component of focal adhesions. Immunofluorescent staining showed that the vinculin expression increased in the group in which hGFs were cultured in CM-Zn/CS/Gel (*p* < 0.05) (Fig. 7A, B). Previously, Rout et al. found that exposure to TGF-β enhanced vinculin expression in fibroblasts to improve adhesion apparatus formation, while the direct influence of TGF-β on vinculin expression has not been extensively studied [44]. In our study, more TGF-β secretion was detected for macrophages cultured on Zn/CS/Gel, from which CM is likely to induce higher vinculin expression in fibroblasts. Moreover, previous studies have also shown that the secretion of pro-inflammatory cytokines by M1 macrophages, such as TNF-α, IL-1β, and IL-6, compromised the expression and activity of focal adhesion proteins, including vinculin [45, 46].

Another important function of fibroblasts in their participation to peri-implant STI is collagen secretion, since collagen fibers are the major components of the tissue matrix around dental implants [47]. Mature tissue matrix can provide a stable scaffold for tissue adhesion, supporting implant-tissue integration. Therefore, we investigated the effect of the immune microenvironment on collagen synthesis of hGFs after culturing cells in CM for 7 days. Immunofluorescent staining indicated that fibroblasts in CM-Zn/CS/Gel showed highest levels of Col I expression (*p* < 0.001) (Fig. 7C, D). An earlier study has reported that TGF-β stimulates Col I expression via the SMAD signal transduction pathway [48]. Regarding to our early observation that macrophages cultured on Zn/CS/Gel exhibit higher TGF-β secretion, we speculate that the CM-Zn/CS/Ge might create a pro-regenerative microenvironment beneficial for collagen deposition.

**Fig. 8:**
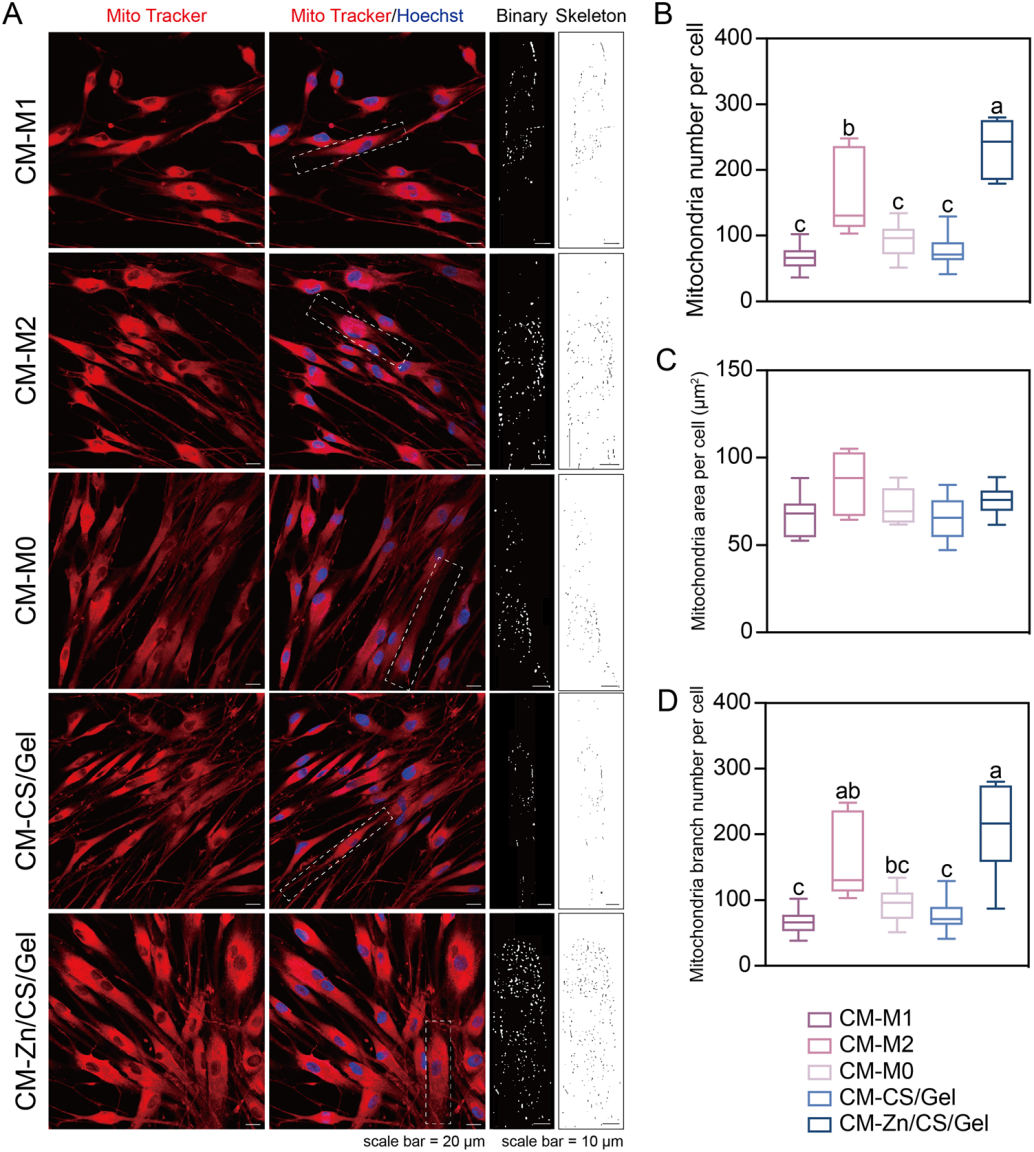
Indirect effects of Zn/CS/Gel coatings on hGF mitochondrial activity. **A** Representative immunofluorescence images and skeleton of mitochondria per fibroblast upon 1-day culture; immunofluorescence image scale bar = 20 μm, skeleton image scale bar = 10 μm; **B** Quantitative analysis of mitochondria number per cell. **C** Quantitative analysis of mitochondrial area per cell. **D** Quantitative analysis of mitochondria branch number per cell. Results indicate that CM-Zn/CS/Gel elevates mitochondrial activity in fibroblasts. a: significance level = 0.05; A: significance level = 0.01

Cellular behavior is highly associated with mitochondrial activity, which can be assessed through mitochondrial number and structure. Previous studies demonstrated that increased mitochondrial activity is correlated to improved cellular metabolism and particular functions such as collagen synthesis [47, 49]. To characterize mitochondrial activity for the various experimental groups, we stained mitochondria in living fibroblasts via MitoTracker (Fig. 8A). Fibroblasts cultured in Zn/CS/Gel conditioned medium showed an increased number of mitochondria (*p* < 0.05) (Fig. 8B, C). The mitochondria branch number in hGFs cultured in conditioned medium Zn/CS/Gel was almost 3 times higher than those from CM-M1 and CM-M0 controls as well as CM-CS/Gel (*p* < 0.01) (Fig. 8D).

As the power source for cellular energy production, mitochondria are of vital importance for maintaining cellular growth and function such as homeostasis and differentiation [49]. Both mitochondrial number and area are critical parameters in mitochondrial biology, as these parameters reflect their function and dynamics. Mitochondria are double-membraned organelles with electron transport chain (ETC) complexes on the inner mitochondrial membrane, responsible for oxidative phosphorylation – the process generating adenosine triphosphate (ATP), the primary energy source for cellular activities. Mitochondrial branching can influence mitochondria distribution and their efficiency in ATP production. A previous study indicated that cells with more branched mitochondria tend to exhibit superior metabolic flexibility, facilitating the adaptation to fluctuating energy demands. The inner mitochondrial membrane is impermeable to most ions, creating an electrochemical gradient or potential across the membrane which is termed as mitochondrial membrane potential (MMP). A former study reported that TGF-β exposure increased the ATP content in podocytes [50]. Their results indicated that TGF-β increases mitochondrial MMP and oxygen consumption rates (OCR), resulting in enhanced reactive oxygen species (ROS) generation via the mammalian target of rapamycin (mTOR) pathway, which might contribute to wound healing. Based on the aforementioned findings, we postulate that the elevated mitochondrial activity in cells treated by CM-Zn/CS/Gel is likely attributed to the higher concentration of TGF-β.

The immunomodulatory impact of zinc in tissue regeneration has gained considerable attention, particularly due to its potential in influencing macrophage polarization states [17]. However, the application of Zn^2+^-containing materials to modulate the immune microenvironment in peri-implant tissue, particularly for STI remains unclear. Consequently, our study aimed to address this gap by incorporating Zn^2+^ into a dental abutment coating and investigating their direct effects on THP-1 derived macrophages and indirect effects on fibroblast behavior.

Compared with previous studies, we adopted a straightforward and cost-effective approach to study the effect of Zn^2+^ on macrophage responses, directly relevant to peri-implant wound healing. The results from ion release kinetics revealed that our Zn/CS/Gel coating exhibits pH-responsive behavior, with increased and expedited release of Zn^2+^ observed under acidic conditions. In combination with the findings that demonstrate the ability of the Zn^2+^-containing coating to stimulate pro-regenerative M2 macrophage polarization, we conclude that these coatings hold strong promise to reduce inflammation and enhance tissue repair around dental implant abutments.

In addition, the healing of tissues around different dental implant components involves complex processes that include various cell types, such as blood cells, immune cells, mesenchymal stem cell and endothelial cells. Previous studies have introduced numerous zinc-based dental materials owing to their osteoimmunological properties [33, 34, 42]. However, no studies have been conducted to investigate the effect of zinc-based immunomodulation on soft tissue healing around dental implant abutments. Our approach to use abutment surface modification with zinc to modulate favorable macrophage polarization with subsequent stimulatory effects on primary gingival fibroblasts offers the opportunity to enhance control over STI. While current *in vitro* evidence is promising, precisely designed *in vivo* studies are required to confirm the immunomodulatory effectiveness of the zinc-containing coatings for clinical application. In addition, the angiogenic effect with the Zn/CS/Gel coating was not investigated in this research. However, according to previous studies, Zn^2+^ has been reported to improve angiogenesis during wound healing process, requiring further research about the angiogenic effect of the Zn/CS/Gel coating [51, 52].

## Conclusion

In this study, we explored the response of macrophages to Zn^2+^, and subsequently studied the macrophage-mediated immunomodulatory effects of Zn^2+^ on gingival fibroblasts. Macrophage morphological and secretome analysis showed that Zn^2+^ effectively stimulates macrophage polarization toward the M2 phenotype at a concentration of 37.5 μM. Incorporation of Zn^2+^ into Zn/CS/Gel resulted in effective pH-responsive Zn^2+^ release to evoke M2 macrophage polarization. Indirect cultures of gingival fibroblasts in macrophage CM demonstrated enhanced adhesion, proliferation, and collagen secretion. In view of the pivotal role of immunomodulation in tissue integration, Zn/CS/Gel coatings hold strong promise to modulate macrophage polarization, thereby stimulating fibroblast response favorable for enhanced peri-implant STI.
